# The Neuroanatomical Organization of Projection Neurons Associated with Different Olfactory Bulb Pathways in the Sea Lamprey, *Petromyzon marinus*

**DOI:** 10.1371/journal.pone.0069525

**Published:** 2013-07-29

**Authors:** Warren W. Green, Alfred Basilious, Réjean Dubuc, Barbara S. Zielinski

**Affiliations:** 1 Department of Biological Sciences, University of Windsor, Windsor, Ontario, Canada; 2 Groupe de Recherche sur le Système Nerveux Central, Département de Physiologie, Université de Montréal, Montréal, Québec, Canada; 3 Groupe de Recherche en Activité Physique Adaptée, Département de Kinanthropologie, Université du Québec à Montréal, Montréal, Québec, Canada; School of Biomedical Sciences, The University of Queensland, Australia

## Abstract

Although there is abundant evidence for segregated processing in the olfactory system across vertebrate taxa, the spatial relationship between the second order projection neurons (PNs) of olfactory subsystems connecting sensory input to higher brain structures is less clear. In the sea lamprey, there is tight coupling between olfaction and locomotion via PNs extending to the posterior tuberculum from the medial region of the olfactory bulb. This medial region receives peripheral input predominantly from the accessory olfactory organ. However, the axons from olfactory sensory neurons residing in the main olfactory epithelium extend to non-medial regions of the olfactory bulb, and the non-medial bulbar PNs extend their axons to the lateral pallium. It is not known if the receptive fields of the PNs in the two output pathways overlap; nor has the morphology of these PNs been investigated. In this study, retrograde labelling was utilized to investigate the PNs belonging to medial and non-medial projections. The dendrites and somata of the medial PNs were confined to medial glomerular neuropil, and dendrites of non-medial PNs did not enter this territory. The cell bodies and dendrites of the non-medial PNs were predominantly located below the glomeruli (frequently deeper in the olfactory bulb). While PNs in both locations contained single or multiple primary dendrites, the somal size was greater for medial than for non-medial PNs. When considered with the evidence-to-date, this study shows different neuroanatomical organization for medial olfactory bulb PNs extending to locomotor control centers and non-medial PNs extending to the lateral pallium in this vertebrate.

## Introduction

The brain contains numerous examples of subsystem pathways for sensory input that enable the perception of complex sensory information and allow for different functional aspects of a stimulus to be processed simultaneously [1], [2], [3]. An example is seen in the olfactory system of mammals, where subtypes of sensory neurons located in the main olfactory epithelium project to specific regions of the olfactory bulb, and sensory neurons in the vomeronasal organ project to the accessory olfactory bulb [4]. The receptive fields of the second order projection neurons (PNs) located in the olfactory bulb and the accessory olfactory bulb are important for gathering synaptic information, and dendritic morphology is an important indicator of synaptic interactions. If dendritic territories of PNs overlap, then these cells likely process similar information.

**Figure 1:**
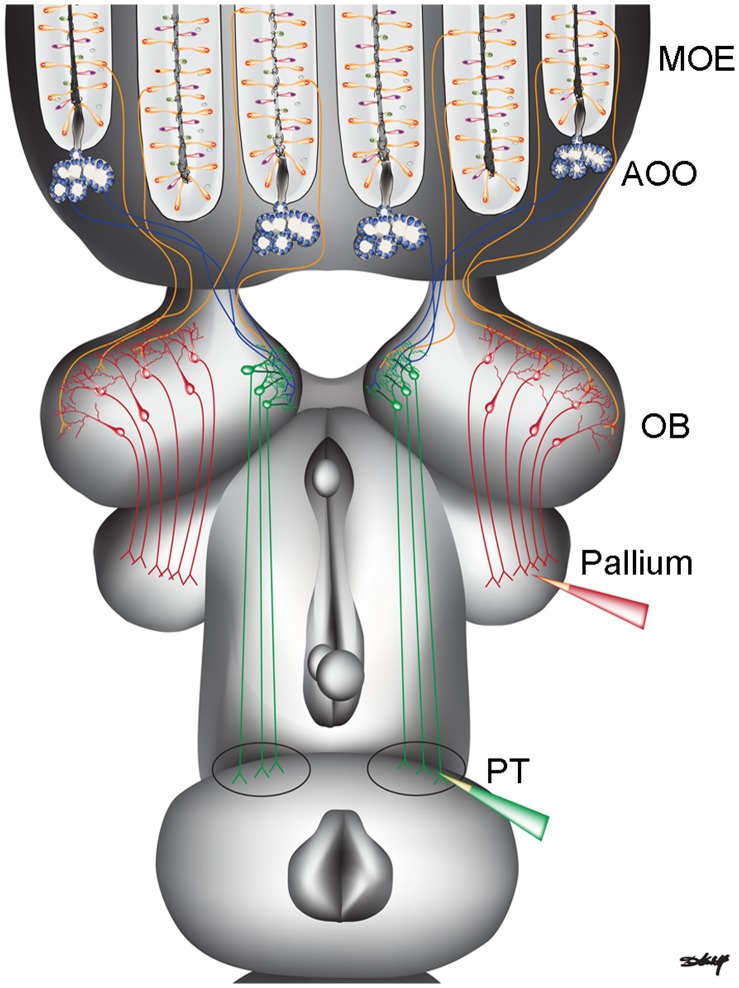
Input and output pathways of projection neurons in the sea lamprey olfactory bulb. These neuronal projections are based on Ren et al [5] and Derjean et al [10]. The medial region of the olfactory bulb receives inputs from the accessory olfactory organ (AOO – blue) as well as sparse inputs from the main olfactory epithelium (MOE – orange). The medial projection neurons (green) project their axons to the posterior tuberculum (PT). The non-medial region of the olfactory bulb receives inputs from the main olfactory epithelium and the non-medial projection neurons (red) project their axons to the pallium. Red and green pipettes indicate location of biocytin insertion to retrogradely label projection neurons in the olfactory bulb (OB).

Recently, a neural substrate underlying the transformation of olfactory inputs to locomotor output was described for the first time in a vertebrate, in the sea lamprey (*Petromyzon marinus*) [5], a model species for studying the neural control of locomotion. Olfactory cues evoke chemotactic responses, especially during the migratory and spawning adult stages when locomotor responses to pheromones are of primary importance [6], [7]. Olfactory sensory input is conducted by PNs in the medial region of the olfactory bulb to the posterior tuberculum, located in the ventral diencephalon (Fig. 1). From this region, the inputs project down to the mesencephalic locomotor region (MLR), known to control locomotor activity (for review see [8], [9]). The MLR initiates locomotion by acting on brainstem motor command cells, the reticulospinal neurons, which directly activate the locomotor networks of the spinal cord. The sensory inputs to the medial region of the olfactory bulb originate predominately from the accessory olfactory organ [10], and are biochemically distinct from sensory neurons projecting from the main olfactory epithelium to non-medial regions of the olfactory bulb [11]. Moreover, PNs in non-medial regions of the olfactory bulb project to the lateral pallium and other forebrain structures [5].

There are also examples of other nerve fiber trajectiories in the olfactory system of lampreys [12], [13], [14]. In *Ichthyomyzon bicuspis* and in *Lampetra planeri*, extra-bulbar fibers project from the olfactory epithelium to the posterior tuberculum [12], [14]. These nervus terminalis or extra-bulbar fibers are unlikely to participate in the locomotor effects observed in *P. marinus* by Derjean et al [5], since the locomotion was induced when glutamate was injected into the medial portion of the olfactory bulb; but glutamate injection in the lateral bulbar region had no locomotor effects. This indicates that the locomotor responses are elicited by activation of somata and/or dendrites in the medial region of the olfactory bulb. While projections from the caudal-lateral region of the olfactory bulb extended to the posterior tuberculum in *I. bicuspis* [12]; tracer loading to the posterior tuberculum of *P.marinus* labeled fibers in the medial, but not the lateral region of the olfactory bulb [5].

While the medial and non-medial PNs in the sea lamprey olfactory bulb project to different targets [5], it is unknown if these two populations share areas of synaptic input or similarities in dendritic morphology. In the olfactory bulb of the lamprey, the cell bodies of mitral cell projection neurons do not form a distinct layer and are dispersed between as well as beneath glomeruli [15], [16], [17]. Multiple primary dendrites extend from the cell body into glomerular neuropil and lateral dendritic branches are absent [16]. The receptive fields and dendritic morphology may undergo changes at different stages in the sea lamprey life cycle, which includes a complex metamorphosis from larvae to young adult prior to the migratory and spawning adult phase [18].

The objective of this study was to investigate the sea lamprey olfactory bulb for distribution and morphology of the dendrites and the somal position of PNs that transmit olfactory inputs to locomotor centers (projections from the medial olfactory bulb to the posterior tuberculum [5]), to compare these with the non-medial PNs that connect to the lateral pallium; and to look for clues of spatial boundaries imposed on the PNs at two different life stages. Since retrograde neuronal labelling was utilized, we could not ensure that only a single projection neuron type was labelled (e.g. mitral or tufted) and therefore we have designated the labelled cells as PNs.

## Materials and Methods

### Ethics Statement

All housing conditions and experimental procedures were in compliance with the Canadian Council on Animal Care. All protocols were approved by the University of Windsor Animal Care Committee (AUPP # 05-13 and 11-02). Special care was taken to minimize the number of animals used in this study and all efforts were made to minimize suffering.

### Experimental Animals

Young adult (post-metamorphic stage seven/downstream migrants) and spawning-phase adult sea lamprey (*Petromyzon marinus*) were supplied by the Great Lakes Fishery Commission and the Department of Fisheries and Oceans Canada. All spawning phase adult sea lamprey were female and the sex of young adult lamprey was not determined. All animals were housed at the University of Windsor in aquaria at 7°C ±1°C in dechlorinated water under static renewal conditions until used. Young adult and spawning adult sea lamprey weighed 4.2 g ±0.4 and 259.5 g ±6.9 (mean ± SEM) respectively, and measured 12.8 cm ±0.8 and 47.2 cm ±0.8 (mean ± SEM), respectively. Young adult and spawning-phase adult sea lamprey [19] (n = 60) were examined to test for possible differences in the PNs located in the medial and non-medial olfactory bulb regions.

### Cresyl Violet Staining of the Olfactory Bulb

Cresyl violet staining was utilized to identify the OB layers along the rostral-caudal axis, and to view the location of putative PN nuclei. Sea lampreys were euthanized in a 1 g/L solution of tricaine methane sulfonate (MS222, FINQUEL, Ayerst Laboratory, New York) and immediately decapitated. The brain was isolated and fixed in 4% paraformaldehyde in 0.1 M phosphate buffer (PB, pH = 7.4), cryoprotected using a sucrose gradient and serial 14 µm coronal sections were prepared on a cryostat (Leica CM3050 S, Leica Microsystems, Germany), stained with cresyl violet, dehydrated and then cleared.

### Retrograde Tracing of Olfactory Bulb PNs

Biocytin (B-1592, Invitrogen, Burlington, ON) was utilized here to retrogradely label the cell bodies and dendrites of PNs extending from the olfactory bulb. Biocytin’s relatively small molecular weight (372 kDa) makes it useful when labelling fine processes. It was previously used as an anterograde and retrograde tracer [20], and as a retrograde label in the sea lamprey [10], [21], [22]. The biocytin was dissolved in deionized water, and then allowed to dry on a slide. The remaining biocytin crystals were applied onto the tip of tungsten probes (tip diameter = 1 µm; 501316, World Precision Instruments, Sarasota, FL) for loading into brain tissue. Sea lampreys were anaesthetized in a 100 mg/L solution of tricaine methane sulfonate (MS222, FINQUEL, Ayerst Laboratory, New York), decapitated, and dissected in lamprey Ringer’s solution. The meninges were removed from the surface of the olfactory bulb, since endogenous expression of avidin binding sites was seen in the meninges in negative controls that were labeled with streptavidin, but did not receive an insertion of biocytin (data not shown). The region between adjacent olfactory bulbs was inaccessible for removing meninges; therefore a small amount of meninges remained on the medial surface of the olfactory bulb. Derjean et al. (2010) [5] showed that the axons of PNs in the medial region of the olfactory bulb terminate in the ventral diencephalon, specifically, the posterior tuberculum (Fig. 1). Accordingly, these PNs were labeled by inserting biocytin crystals into the ventral diencephalon including the posterior tuberculum. Since axons of non-medial PNs project to and terminate in the lateral pallium [5], these PNs were labeled by cutting the pallium and inserting biocytin crystals into the lateral pallium (Fig. 1). The brain was then incubated in cold oxygenated Ringer’s solution for 8 hours to allow biocytin to move retrogradely in the axons and label PN cell bodies and dendrites. Following incubation, the tissue was fixed in 4% paraformaldehyde in 0.1 M phosphate buffer (PB, pH = 7.4). The tissue was then cryoprotected using a sucrose gradient (10%, 20%, then 30% sucrose in 0.1 M phosphate buffered saline; PBS) and cryosectioned (Leica CM3050 S, Leica Microsystems, Germany) in serial 35 µm coronal sections. These were rehydrated in 0.1 M PBS and then placed in Alexa Fluor 568 streptavidin (S11226∶1:200 Molecular Probes, Eugene, OR) in 0.1 M PBS overnight at 4°C to visualize the biotinylated PNs. Slides were rinsed with 0.1 M PBS 3 times for 10 minutes and coverslipped using fluoromount-G (cat. # 0100-01, Southern Biotech, Birmingham, AL).

### GS1B_4_ Labelling of Olfactory Glomeruli

The axons of olfactory sensory neurons projecting into the glomeruli of the olfactory bulb (both medial and nonmedial) can be visualized by labelling with *Griffonia simplicifolia* lectin I, isolectin β4 (GS1β_4_) [11], [23]. The GS1β_4_ binds to galactosyl residues present on the axons of lamprey olfactory sensory neurons [23], [24]. It was utilized to show the spatial relationship between retrogradely labelled PNs and the axons of olfactory sensory neurons. After the biocytin filled PNs had been labelled with Alexa Fluor Streptavidin 568, these slides were placed into Fluorescein 488 GS1β_4_ (10 µg/ml, FL-1201, Vector Laboratories, Burlington, ON) and incubated overnight at 4°C. Slides were then rinsed in 0.1 M PBS (3 rinses, 10 minutes each) and coverslipped.

### Microscopy

Tissue sections were observed on a Nikon Eclipse E600 fluorescence microscope (Nikon, Tokyo, Japan) and images were archived with a Q-Imaging Retiga 1300 digital camera (Q-Imaging Corporation, Burnaby, B.C., Canada) using Northern Eclipse software (EMPIX Imaging Inc., Mississauga, Ontario, Canada). Confocal microscopy (Olympus FluoView FV1000 FV-10 ASW, Olympus, Tokyo, Japan) was used to investigate the morphology of PNs, including the dendritic arborizations.

### Morphometry

The volume of non-medial and medial glomeruli, as well as the number of medial PNs and the soma size of non-medial and medial PNs was determined in young adult (n = 4) and spawning adult (n = 4) lampreys following injection of biocytin into the posterior tuberculum and labelling of glomeruli using GS1β_4_ lectin.

Glomeruli were identified in the olfactory bulb as spheroidal structures that were labelled with GS1β_4_. Micrographs were taken of serial sections containing labelled glomeruli along the rostral-caudal extent of the olfactory bulb. The area of glomeruli were determined by manually tracing their circumference using the freehand drawing tool in Image J. The area values were subsequently multiplied by the section thickness and the number of sections to determine the volume of non-medial glomeruli and the medial glomerulus. Initially, the glomerular volume was determined for both the left and right olfactory bulbs and was found to not differ significantly (paired t-test, t_2_ = −0.4361, P = 0.7053)(Sigmastat, Systat software Inc), therefore, only volume values for the right olfactory bulbs were subsequently determined. Two-sample t-tests were utilized to determine whether the percentage of total glomerular volume occupied by the medial glomerulus or non-medial glomeruli differed between young adult and spawning adult lamprey (Sigmastat, Systat Software Inc).

The number of PNs in the medial region was counted in young adult (n = 4) and spawning adult (n = 3) lamprey using Northern Eclipse software (EMPIX Imaging Inc., Mississauga, Ontario, Canada). Cell counts were performed using the physical dissector method whereby the number of PNs in a given section were counted and subsequently compared with the preceding section to ensure that no cell body was counted twice. A two-sample t-test was utilized to determine whether the number of medial PNs differed between young adult and spawning adult lamprey (Sigmastat, Systat software Inc).

The diameter of PN cell bodies on their major (longest) axis was measured using Northern Eclipse software (EMPIX Imaging Inc., Mississauga, Ontario, Canada). We measured only PN cell bodies that were visible in their entirety by focusing through sections with a 40×oil immersion objective (NA 1.3). The length of the major axis (diameter along the longest axis) was measured in the plane of focus where the cell was largest. A total of 50 PNs were selected in medial (n = 5) and non-medial labelled (n = 5) olfactory bulb tissue in both young adult and spawning adult sea lamprey. Values reported for PNs cell body diameters are mean ± SEM. Post acquisition analysis of differences in cell body diameter between the medial and non-medial regions of the olfactory bulb passed the statistical test for normality but not for equal variance, thus a Kruskal-Wallis One Way Analysis of Variance on Ranks followed by Tukey *post hoc* pairwise-comparisons was used (Sigmastat, Systat software Inc).

The primary dendrites were directed outwardly towards a glomerulus [25] and were differentiated from an axon based on size (dendrites have a thicker diameter than axons [16]). Generally, PN cell dendrites project towards the surface of the olfactory bulb, while axons project towards the centre of the olfactory bulb [16]. We utilized nomenclature defined by Fuller et al. (2006) [26] for designating the dendritic composition of the PNs as unidendritic (possessing a single primary dendrite) or multidendritic (with two or more primary dendrites). Tallies of PNs (unidendritic and multidendritic) were performed utilizing two young adult and two spawning adult lamprey for both the medial (10 PNs/tissue; n = 4; 40 PNs total) and non-medial (20 PNs/tissue; n = 4; 80 PNs total) regions of the olfactory bulb. Only PNs that included the entire cell body and at least the proximal portion of these neuronal processes within a tissue section were assessed as unidendritic or multidendritic. Moreover, the classification of PNs as unidendritic or multidendritic was established by through-focus examination and confocal microscope z-stack reconstruction of these cells. In the confocal z-stacks, each section was examined from the proximal portion of the dendrite at the cell body to the distal end of the dendrite to ensure the continuity of the dendrites.

## Results

**Figure 2:**
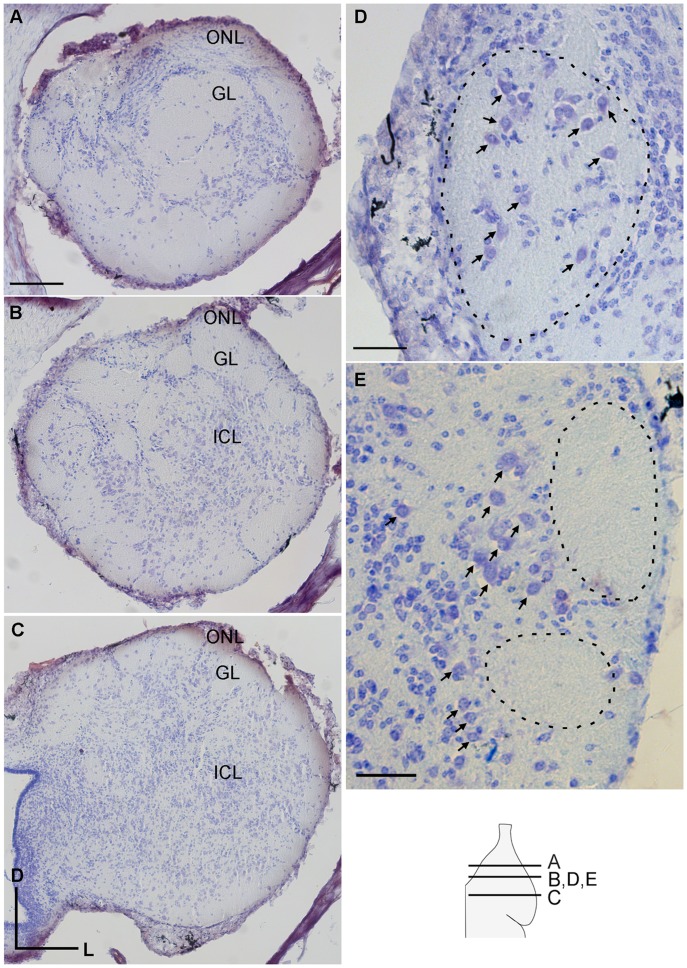
A survey of the olfactory bulb in young adult sea lamprey. Cresyl violet stained coronal sections show the olfactory nerve layer (ONL), glomerular layer (GL), and the internal cell layer (ICL) along the rostral –caudal extent of the sea lamprey olfactory bulb (A–C). Scale bar, shown in A (200 µm) is the same for images A–C. High magnification view of large nuclei in the glomerulus in the medial region (D) and in the internal cell layer in the non-medial region (E). Scale bars in D and E are 50 µm. Dashed lines in D and E outline the location of the medial and non-medial glomerular neuropil, respectively. Arrows point to large nuclei. Inset in bottom right corner is a diagram of the olfactory bulb in the horizontal plane with lines representing the location of the coronal sections A–E.

The olfactory nerve layer and the glomerular layer of the olfactory bulb were seen in rostral coronal views of Nissl stained preparations (Fig. 2A). In more caudal sections, where the internal cell layer appeared below the glomeruli (Fig. 2 B, C), differences between medial and non-medial glomerular territories were evident, as large nuclei were prominent within medial glomerular neuropil (Fig. 2 D), but not in the other glomeruli. Instead, large nuclei were concentrated in the internal cell layer below the non-medial glomeruli (Fig. 2E).

### Localization of Olfactory Bulb PNs

**Figure 3:**
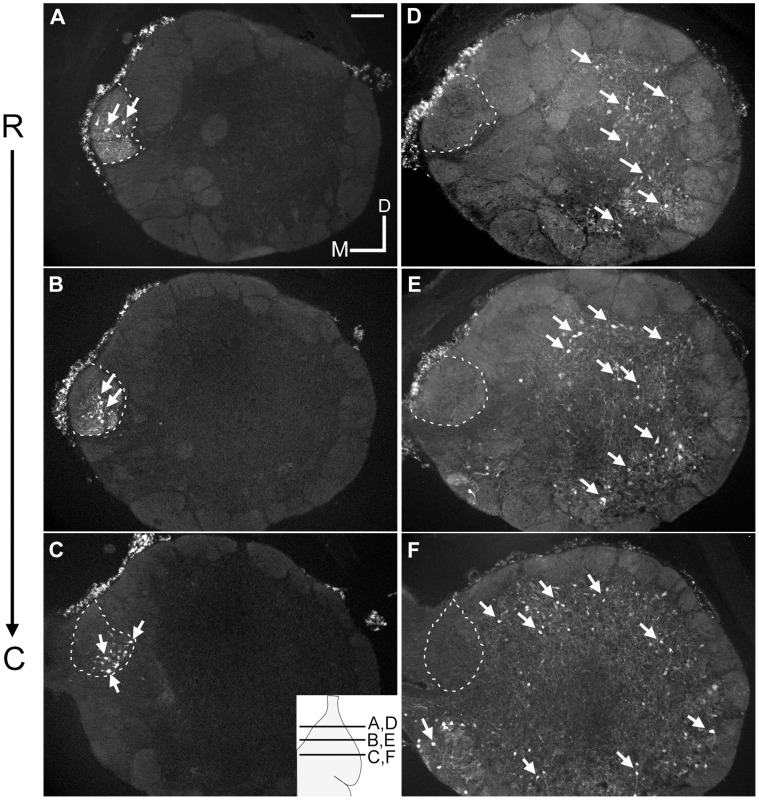
Distribution of medial and non-medial projection neurons. Rostral-caudal distribution of retrogradely labeled PNs in coronal sections of the young adult olfactory bulb following insertion of biocytin into the posterior tuberculum (A–C) or the lateral pallium (D–F). Biocytin insertion into the posterior tuberculum exclusively labelled medial PNs (A–C), while biocytin insertion into the lateral pallium labelled non-medial PNs (D–F). Scale bar, shown in A (125 µm) is the same for all images. Inset in C is a diagram of the olfactory bulb in the horizontal plane with lines representing the location of the coronal sections. Dashed lines in A–F outline the location of the medial glomerular neuropil. Arrows point to retrogradely labeled cell bodies.

**Figure 4:**
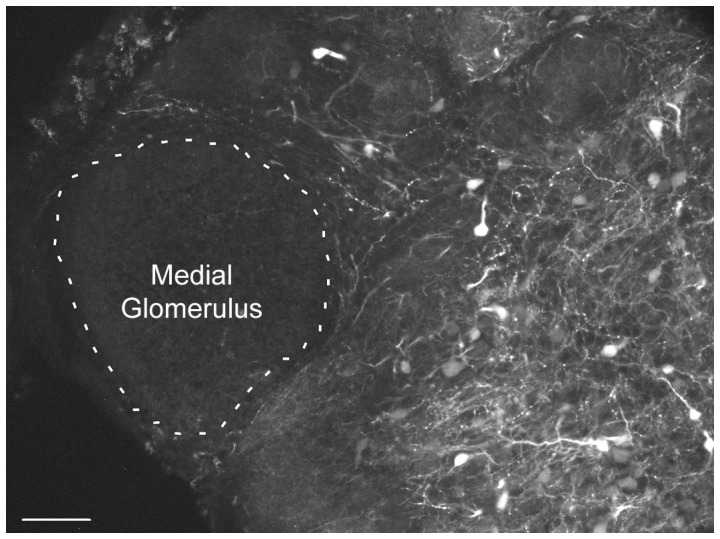
Medial projection neurons do not project axons to the lateral pallium. Non-medial projection neurons (PNs) retrogradely labeled from the lateral pallium did not enter the medial glomerulus. Dashed line denotes the boundary of the medial glomerulus. Scale bar is 60 µm.

**Figure 5:**
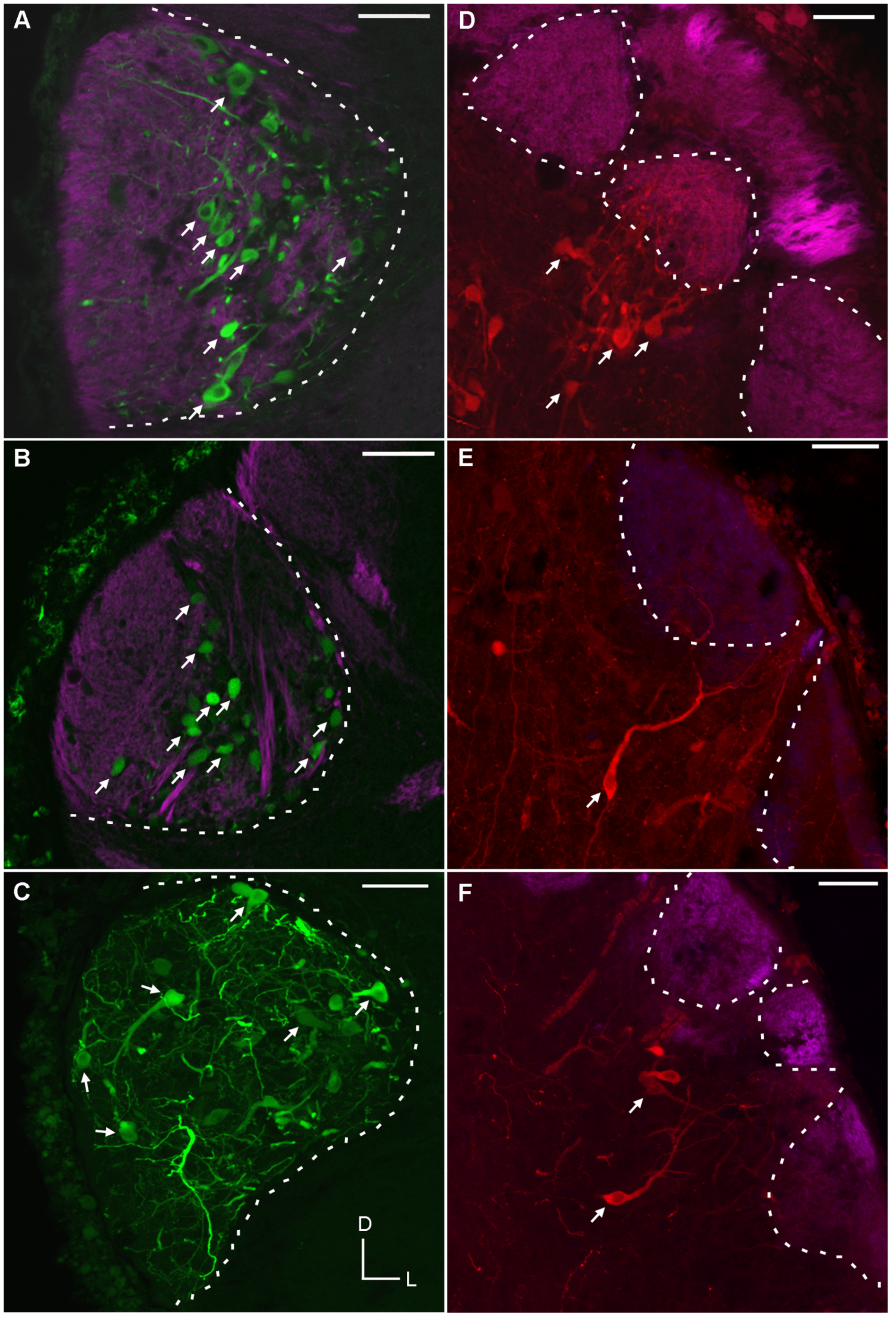
Medial projection neurons are located within the glomerular layer. The axons of olfactory sensory neurons were labeled with *Griffonia simplicifolia* Lectin I, isolectin β4; (purple). The cell bodies (A–C) and dendrites (C) of retrogradely labelled medial PNs (green) are located within the glomerular neuropil. For non-medial retrogradely labeled PNs (red), the cell bodies were observed proximal (deep) to the glomerular layer (D–F), and dendrites projected into the non-medial glomeruli (E, F). Scale bars shown in A–F are 50 µm. Arrows point to retrogradely labelled cell bodies. Dashed lines denote the boundaries of glomeruli.

Biocytin, inserted into the ventral diencephalon, retrogradely labeled medial PNs along the rostral-caudal extent of the medial glomerular neuropil, without labeling cells in other regions of the olfactory bulb (Figure 3; A–C). Non-medial PNs were labeled following biocytin application to the lateral pallium (Figure 3; D–F); and the dendrites of these non-medial PNs did not extend into the medial glomerulus (Fig. 4). Axonal fibers extended caudally and were traced back to the biocytin injection site. In both young adult and spawning adult stages, the dendrites and cell bodies of the medial PNs were confined to glomerular neuropil containing the axons of GS1B_4_ lectin labelled olfactory sensory neurons (Figure 5A–C). The boundary of this medial glomerulus was clear, as it contained the cell bodies and processes of PNs retrogradely labeled from the posterior tuberculum. However, for non-medial PNs, the somal position was outside (often proximal) to glomeruli, and dendrites extended into glomerular neuropil (Figure 5 D–F).

The volume was greater in the spawning adult lamprey than in the young adult lamprey for non-medial (t-test, t_(6)_ = −5.6506, P = 0.0013), medial (t-test, t_(6)_ = −2.6713, P = 0.0370), and total (t-test, t_(6)_ = −5.5257, P = 0.0015) glomeruli (Table 1). Although the total glomerular volume of the olfactory bulb was approximately 4.3 X larger in the spawning adult compared to the young adult (post metamorphic) stage, the position and relative percent glomerular volume occupied by the medial glomerulus remained similar in both life stages (t-test, t_(6)_ = −1.7439, P = 0.1318). Likewise, the relative percent glomerular volume occupied by the non-medial glomeruli was similar in the young adult and the spawning adult stages (t-test, t_(6)_ = −1.7434, P = 0.1319) (Table 1). These measurements indicate the bulbar volume occupied by the medial glomerulus and the non-medial glomeruli increased proportionately with the total glomerular volume of the olfactory bulb, from the young adult to the spawning adult stage.

**Table 1:** Morphometric characteristics of the olfactory bulb and projection neurons in young and spawning adult lamprey.

	Young Adult	Spawning Adult
Volume of non-medial glomeruli (µm^3^)	1.21E+08±3.11E+06 (4)†	5.40E+08±7.41E+07 (4)
Volume of medial glomerulus (µm^3^)	1.28E+07±3.14E+06 (4)†	3.11E+07±6.08E+06 (4)
Total glomerular volume (µm^3^)	1.34E+08±1.92E+06 (4)†	5.72E+08±7.92E+07 (4)
Percentage of total glomerular volume occupied by medial glomerulus	9.5±2.3 (4)	5.4±0.6 (4)
Percentage of total glomerular volume occupied by non-medial glomeruli	90.45±2.28 (4)	94.56±0.60 (4)
Number of labeled medial PNs	95±3.5 (4)	85.7±1.8 (3)
Soma Size of medial PNs (µm)	14.05±1.5 (50)* †	19.56±1.8 (50)*
Soma Size of non-medial PNs (µm)	12.56±0.9 (50)†	15.32±1.9 (50)

Volume of olfactory bulb glomeruli, projection neuron (PN) cell counts, and the soma size of PNs in the olfactory bulb of young adult (post metamorphic) and spawning adult sea lamprey. All values are mean ± SEM. Sample size is indicated in brackets for each category.

† denotes a statistically significant difference (p<0.05) between young adult and spawning adult lamprey while an asterisk.

* denotes a statistically significant difference (p<0.001) between medial and non-medial PN soma size within a given life stage.

### Tallies and Size of PN Somata

While the medial glomerulus was larger in the spawning adult stage compared to the young adult stage (Table 1), the estimated number of retrogradely labeled PNs located within the medial glomerulus in young adult and spawning adult lamprey was 95±3.4 and 85.7±1.8 (mean ± SEM), respectively (Table 1). Although there were slightly more retrogradely labeled PNs in the medial glomerulus of young than spawning adults, this difference was not significant (t-test, t_(5)_ = 2.0917, P = 0.091). In the spawning adult stage, the nuclei were concentrated in the caudal portion of the medial glomerulus, and the rostral portion largely contained the retrogradely labeled dendrites.

In spawning adult lamprey, the cell body diameter of medial PNs was 19.56±1.82 µm (mean ± SEM) compared to 15.32±1.93 µm (mean ± SEM) in non-medially located PNs (Kruskal-Wallis, H_3_ = 137.5, P<0.001) (Table 1). A difference in cell body size was also observed in young adult stage lamprey, as medial PN cell body diameter was 14.05±1.50 µm (mean ± SEM) compared to 12.56±0.90 µm (mean ± SEM) in non-medial PNs (Kruskal-Wallis, H_3_ = 137.5, P<0.001) (Table 1). These findings indicate that medial PNs were larger than non-medial PN cell bodies in both young adult and spawning adult lampreys. Moreover, PN cell body size was greater in spawning adult lamprey compared to young adult lamprey for both medial or non-medial located PNs (Kruskal-Wallis, H_3_ = 137.5, P<0.001) (Table 1).

### Projection Neuron Morphology

**Figure 6:**
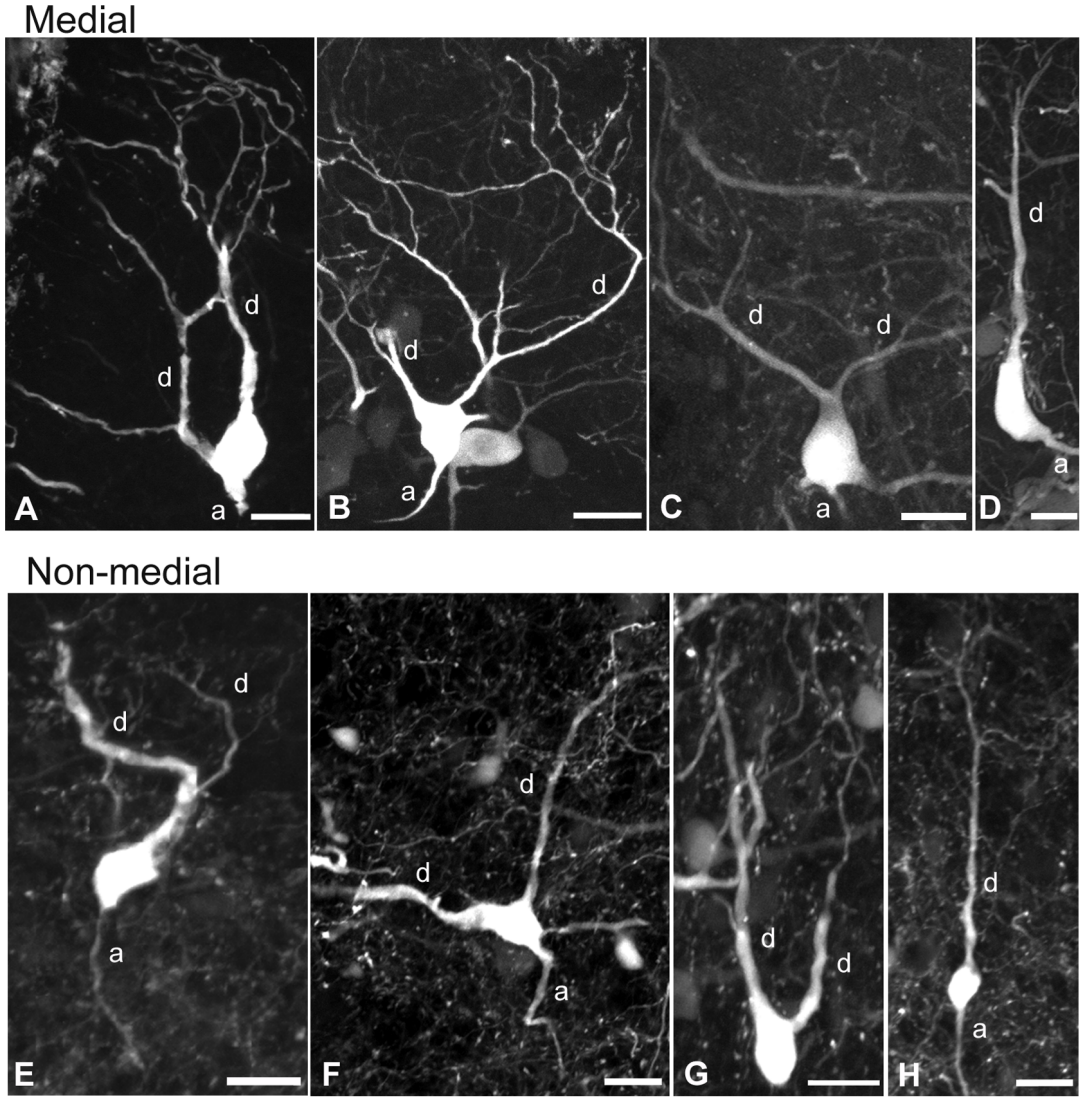
The morphology of olfactory bulb projection neurons. The morphology of medial and non-medial projection neurons (PNs) retrogradely labelled with biocytin in the olfactory bulb of young adult and spawning adult lamprey. Multi-dendritic PNs are shown in the medial (A–C) and non-medial (E–G) region of the olfactory bulb. Unidendritic PNs were observed in the medial (D) and non-medial (H) olfactory bulb. Axons (a) and dendrites (d) of PNs are indicated on each image. Each of the PNs shown were radially oriented, except G, which was tangential. Scale bar is 20 µm.

The morphology of medial (Figure 6 A–D) and non-medial (Figure 6 E–H) retrogradely labelled PNs was similar in many ways. In both locations, the shape of the labelled cell bodies was triangular, ovoid, or elongate. The dendrites were classified as primary dendrites [25] since they extended towards glomeruli, into glomeruli or were situated entirely within glomeruli (in the case of the medial PNs). Secondary dendrites (extending laterally into the granular layer) were absent. Dendrites of medial PNs were oriented radially. Dendrites of non-medial PNs were also radial, although some tangentially oriented dendrites were also observed (Figure 6G). Unidendritic and multidendritic PNs were located in both the medial and non-medial regions (Figure 6). In the non-medial region, 68.75% of the PNs were unidendritic (55/80; n = 4; χ^2^ = 11.25; p<0.001; df = 1); and in the medial region, 62.5% were unidendritic (25/40; n = 4; χ^2^ = 2.5; p>0.10; df = 1). Of the multidendritic PNs observed, a larger proportion was located in the medial region (37.5%; 15/40, n = 4) compared to the non-medial region (31.25%; 25/80, n = 4).

## Discussion

The present study investigated whether there are differences in the distribution and morphology of PN soma and dendrites in the medial and non-medial regions of the olfactory bulb in two life stages of the sea lamprey. In the medial region, the somata and dendrites of PNs were located entirely within the medial glomerulus, and somata were larger than those of PNs in the rest of the olfactory bulb. In contrast, the somata of non-medial PNs were located below the glomerular layer. In addition, both the medial and non-medial olfactory bulb regions exhibited heterogeneous soma and dendrite morphology. The relative volume of the region occupied by the cell bodies and dendrites of the medial PNs was similar in both young adult (post metamorphic) and spawning adult stages. The post metamorphic stage leads directly to the parasitic feeding stage that moves in response to the odours of prey [27]; whereas the migratory adult and spawning adult lamprey show movement responses to pheromones [6], [7], [28].

The medial glomerular neuropil is the site of peripheral axonal projections from the accessory olfactory organ [10]. The location of soma and dendrites of medial PNs entirely within this region suggests that these second order neurons recruit synaptic information from the accessory olfactory organ. Potentially, neural signals processed in the medial glomerulus are not influenced directly by neural activity in the non-medial regions, the site of axon terminals from the olfactory sensory neurons from the main olfactory epithelium [10]. Moreover, there will be a large input effect and the space constant will likely be small for the medial PNs, since both the soma and dendrites are able to come into contact with synaptic terminals entirely within the medial glomerulus. Likely, the medial PNs are utilized during both the young adult and the spawning adult stage life stages, as the size of this glomerulus increased proportionately, along with the size of the non-medial glomeruli, and the number of labeled medial PNs did not change significantly between the two stages.

While PNs in the medial and non-medial regions of the olfactory bulb have segregated receptive fields, they share common soma shapes and dendrite morphologies that are consistent with those previously observed in lamprey. In *Lampetra japonica,* soma were located around glomeruli, had spindle, fusiform, triangular, or polygonal shape and had two or more primary dendrites and no secondary dendrites [16]. Multiple primary dendrites extending radially into multiple glomeruli are common for mitral cells in the olfactory bulb of teleost fish [26], [29], [30], [31] and in the mammalian accessory olfactory bulb [3], [32], [33]. The soma shape of the PNs investigated in the present study were triangular, ovoid, or elongated and generally matched the morphology of mitral cells in the Arctic lamprey [16]. While Iwahori (1987) [16] identified only multidendritic mitral cells (i.e. PNs) in *L. japonica*, by using a Golgi stain, we saw both unidendendritic and multidendritic by using a retrograde labelling approach. Unidendritic mitral cells have previously been seen by retrograde labelling in the olfactory bulb of zebrafish [26]. In accordance with observations of mitral cells in zebrafish [26], a greater proportion of multidendritic PNs were located in the medial region and soma size for medial PNs was greater than for non-medial PNs in the sea lamprey as well. Regional differences in PN morphology exists in the tench (*Tinca tinca*), a teleost fish. Two types of mitral cells (based on soma) were differentially distributed in the medial and lateral regions of the olfactory bulb [29]. Likewise, in the moth (*Bombyx mori*) antennal lobe - PNs innervating the macroglomerular complex (responding to pheromones) did not have extraglomerular processes, while those innervating ordinary glomeruli did [34]. These differences in size and dendrite morphology between the medial and non-medial PNs further supports the idea of two populations of bulbar PNs, and reflects a trend that is also seen in teleosts [27], [31]. Moreover, these morphological differences in PNs along segregated processing pathways may have functional implications.

The organization of the non-medial lamprey olfactory bulb is similar to that of teleost fish, whereby PN soma are located in the internal cell layer and extend their dendrites up into the glomerular layer [31]. The olfactory bulb of mammals is a highly laminar structure compared to that of teleosts. Mitral/tufted cell (PN) soma reside in the deeper mitral cell and external plexiform layers, respectively, and posses a single primary dendrite which extends radially from the cell body up into a single glomerulus as well as laterally branching secondary (basal) dendrites that extend horizontally [3], [25]. In both teleosts and mammals, lateral inhibition between PNs innervating separate glomeruli is thought to play a central role in odour information processing via axon collaterals or basal dendrites, respectively [30], [35], [36]. Since the non-medial region of the lamprey olfactory bulb exhibits a similar organization to that of teleosts, similar PN circuitry may exist.

In contrast, PN soma and dendrites in the medial region of the lamprey olfactory bulb were confined entirely to inside a single glomerulus and did not have dendrites extending into other layers or regions of the olfactory bulb. In this way, the medial PNs may be similar to external tufted cells in the mammalian olfactory bulb, which reside in the glomerular layer, surround glomeruli, and are not regulated by inhibitory inputs from granule cells [3], [37]. Due to the confined nature of the medial PNs, and presumably any associated interneurons, within a single glomerulus, it may be possible that a local self-inhibitory circuit [38] is involved in odour processing within the medial olfactory bulb. In such a circuit, input from olfactory sensory neurons to the medial glomerulus would generate inhibition within the glomerulus via local interneurons [38].

The findings of the current study along with those of Derjean et al [5], Ren et al [10] and Frontini et al [11] suggest that the sea lamprey olfactory bulb contains medial and non-medial olfactory pathways. These two pathways are likely not perfectly segregated, as previous studies of lampreys have shown different projections linking the olfactory system and the posterior tuberculum [12], [13].

Olfactory subsystems have been observed in many other organisms. The main olfactory epithelium/main olfactory bulb and the vomeronasal/accessory olfactory bulb subsystems are well known [4]. Recent work showed that mitral and tufted cells in the mammalian main olfactory bulb exhibit parallel pathways that terminate in different regions of the olfactory cortex [39]. Moreover, in teleosts mitral cells in the medial region predominantly display odour responses to bile acids and project axons along the medial olfactory tract, whereas mitral cells in the lateral olfactory bulb predominately respond to amino acids and project axons along the lateral olfactory tract [26], [40], [41], [42], [43]. Although sea lampreys display anatomical differences of the medial and non-medial olfactory bulb, odorant specificity of the medial and non-medial olfactory bulb has yet to be fully elucidated.

The results of the present study demonstrate that the medial region of the olfactory bulb in the sea lamprey is an anatomically defined and morphologically distinct region that seems to be isolated from the non-medial region of the olfactory bulb. Previous work has demonstrated that the medial olfactory bulb of sea lamprey has a unique output pathway to locomotor command centres [5] and is the only site of sensory input from the accessory olfactory organ [10]. The unique neuroanatomical features described here suggest that the medial region may be not only anatomically different, but also functionally different from the non-medial region. Furthermore, this study supports the hypothesis of Derjean et al. (2010) [5] that there are two spatially segregated olfactory pathways originating in the sea lamprey olfactory bulb. Future work in our lab is focused on determining the PN odour response profiles in the medial and non-medial olfactory bulb and how these responses are modulated.
